# Performance of 20 rapid antigen detection tests to detect SARS-CoV-2 B.1.617.2 (Delta) and B.1.1.529 (Omicron) variants using a clinical specimen panel from January 2022, Berlin, Germany

**DOI:** 10.2807/1560-7917.ES.2023.28.16.2200615

**Published:** 2023-04-20

**Authors:** Andreas Puyskens, Fatimanur Bayram, Akin Sesver, Janine Michel, Eva Krause, Daniel Bourquain, Angela Filomena, Katharina Esser-Nobis, Carla Steffanowski, C Micha Nübling, Heinrich Scheiblauer, Lars Schaade, Andreas Nitsche

**Affiliations:** 1 Robert Koch Institute, Highly Pathogenic Viruses, Centre for Biological Threats and Special Pathogens, WHO Reference Laboratory for SARS-CoV-2 and WHO Collaborating Centre for Emerging Infections and Biological Threats, Berlin, Germany; 2 Paul-Ehrlich-Institute, Testing Laboratory for In-vitro Diagnostic Medical Devices, Langen, Germany; 3 Paul-Ehrlich-Institute, Division Major Policy Issues, Coordination, Langen, Germany

**Keywords:** SARS-CoV-2, B.1.617.2 (Delta), B.1.1.529 (Omicron), rapid antigen detection test

## Abstract

**Background:**

There are conflicting reports on the performance of rapid antigen detection tests (RDT) in the detection of the SARS-CoV-2 Omicron (B.1.1.529) variant; however, these tests continue to be used frequently to detect potentially contagious individuals with high viral loads.

**Aim:**

The aim of this study was to investigate comparative detection of the Delta (B.1.617.2) and Omicron variants by using a selection of 20 RDT and a limited panel of pooled combined oro- and nasopharyngeal clinical Delta and Omicron specimens.

**Methods:**

We tested 20 CE-marked RDT for their performance to detect SARS-CoV-2 Delta and Omicron by using a panel of pooled clinical specimens collected in January 2022 in Berlin, Germany.

**Results:**

We observed equivalent detection performance for Delta and Omicron for most RDT, and sensitivity was widely in line with our previous pre-Delta/Omicron evaluation. Some variation for individual RDT was observed either for Delta vs Omicron detection, or when compared with the previous evaluation, which may be explained both by different panel sizes resulting in different data robustness and potential limitation of batch-to-batch consistency. Additional experiments with three RDT using non-pooled routine clinical samples confirmed comparable performance to detect Delta vs Omicron. Overall, RDT that were previously positively evaluated retained good performance also for Delta and Omicron variants.

**Conclusion:**

Our findings suggest that currently available RDT are sufficient for the detection of SARS-CoV-2 Delta and Omicron variants.

**Key public health message**

**What did you want to address in this study?**

We asked whether available antigen tests could detect SARS-CoV-2 Delta and Omicron variants as well as previously circulating virus variants.

**What have we learnt from this study?**

All 20 CE-marked antigen tests used in this study were able to detect Delta and Omicron variants, and most tests did so as accurately as for previous virus variants.

**What are the implications of your findings for public health?**

Our findings suggest that currently available antigen tests are sufficient for the detection of SARS-CoV-2 Delta and Omicron variants.

## Introduction

Rapid antigen detection tests (RDT) have become a central pillar in public health measures worldwide, enabling fast detection of severe acute respiratory syndrome coronavirus 2 (SARS-CoV-2)-infected individuals on the spot in a variety of settings [1,2]. Despite the continued frequent use of SARS-CoV-2 RDT to detect potentially contagious individuals with high viral loads, there are conflicting reports on the performance of such tests in the detection of the SARS-CoV-2 Omicron variant (Phylogenetic Assignment of Named Global Outbreak Lineages (Pangolin) designation B.1.1.529) [3-10]. 

The aim of this study was to investigate comparative detection of Delta (Pangolin designation B.1.617.2) and Omicron variants by using a selection of 20 RDT and a limited panel of pooled oro-/nasopharyngeal clinical Delta and Omicron specimens. Differences in sensitivity observed between RDT were further compared with a previous evaluation of the respective tests based on pre-Delta/Omicron specimens [11,12].

## Methods

### Establishment of a test panel using Delta and Omicron clinical specimens

To characterise RDT performance, we prepared a test panel comprised of four Delta and 11 Omicron sample pools ranging from 2.46 × 10^4^ to 4.84 × 10^6^ and from 3.16 × 10^3^ to 1.23 × 10^7^ genome copies/mL, respectively. Patient samples were collected as dry combined oro- and nasopharyngeal swabs in late December 2021 and January 2022 at different collection sites in Berlin, Germany. Swab samples were shipped at room temperature and received within 24 h after sampling and stored upon arrival at 4 °C over night. The next day, dry swab samples were resuspended in 1 mL of phosphate-buffered saline (PBS), and 140 µL of each resuspended sample were used for subsequent RNA extraction with the QIAamp Viral RNA Kit (Qiagen). Residual swab suspensions were stored at −80 °C until later use for the preparation of specimen pools. Presence of SARS-CoV-2 RNA was confirmed by real-time PCR using the previously published RKI/ZBS1 SARS-CoV-2 protocol [13]. Quantification cycle (Cq) values shown refer to the SARS-CoV-2 envelope (E) gene. Subsequently, SARS-CoV-2 lineages were identified either by variant typing using commercial PCRs (TaqMan SARS-CoV-2 mutation panel, Thermo Fisher Scientific) or whole genome sequencing using nanopore sequencing (Oxford Nanopore Technologies) [14]. Specimens were selected for pooling depending on the identified variant and respective viral RNA concentrations. For preparation of specimen pools, stored swab suspensions were thawed once at room temperature and mixed by vortexing, and supernatants from three up to 10 different individuals were pooled to achieve a final pool volume of 1 mL. Subsequently, these 1 mL pools were further diluted in 4 mL of PBS. Pools were aliquoted as 300 µl aliquots and frozen at −80 °C. The PBS served as negative specificity and inhibition control and was added to the evaluation panel. Lastly, an aliquot of each specimen pool was thawed, 25 µL extracted and again analysed by real-time PCR to determine the final viral RNA concentration of the pool aliquots.

### Selection of rapid antigen detection tests

The study was designed to reveal potential differences in RDT detection performance between Omicron and Delta. The study was limited to investigate an exemplary selection of 20 representative RDT marketed in the European Union (EU), covering a range of sensitivities that passed minimal sensitivity criteria as defined previously (corresponding to a minimum detection rate of 75% of specimen pools with a Cq value ≤ 25) [11,12]. All RDT included in the study targeted the nucleocapsid (N) protein. Test and brand names are not disclosed in this article as underlying data were collected for regulatory purposes. Ranking of RDT sensitivity (based on genome copies/mL) was compared with the ranking of the corresponding tests from earlier evaluations preceding the Delta/Omicron phase of the pandemic [11,12]. Preparation of specimen panels, testing procedures and determination of viral RNA concentrations by real-time PCR were comparable between studies. Differences in sample volumes subjected to RDT between specimen panels depended on viral RNA concentrations of specimen pools and were adjusted accordingly for comparability.

### Testing procedures for rapid antigen detection tests

For RDT testing, aliquots were thawed at room temperature, mixed by vortexing and briefly spun down to collect samples. To achieve comparability of viral RNA concentrations from previous specimen panels, 25 µL per sample pool were used for testing. All swabs provided with the RDT kits and used in this study were able to fully absorb the 25 µL of sample volume. After collection of samples using the respective swabs, all RDT were processed in accordance to the manufacturer's instructions; i.e. the swab was transferred to the test-specific buffer, extracted and the eluate applied in the prescribed volume to the test. Each test result was interpreted independently by two trained laboratory technicians. If results were not in agreement, a third trained laboratory technician independently interpreted the test and the result in favour was noted. All RDT assessed in this study displayed a visible control line during testing. Different RDT were tested with the entire specimen panel on different days. Each RDT was tested once with each specimen pool and negative control. Thawed aliquots were used for testing on the same day and discarded at the end of each day.

## Results

### Performance of rapid antigen detection tests with Delta and Omicron specimen pools

**Figure 1:**
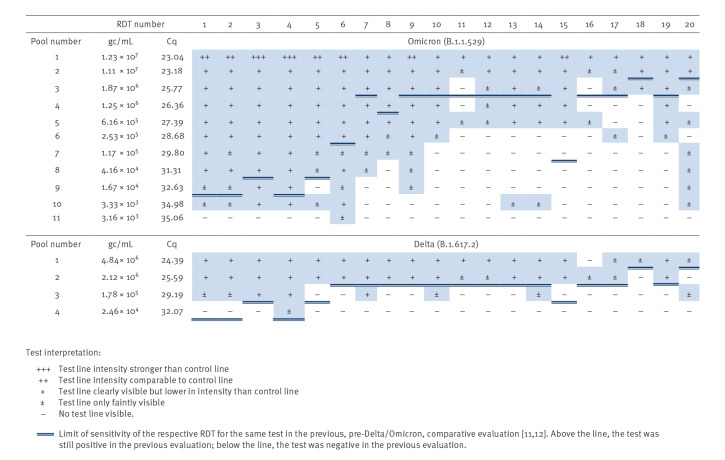
Overview of rapid antigen detection test performance using SARS-CoV-2 Delta and Omicron pooled clinical specimens, Germany, January 2022 (n = 20)

All RDT were able to detect Delta and Omicron variants (Figure 1). Direct comparison of Delta and Omicron showed an overall comparable degree of detection for both variants in the RDT tested. Furthermore, RDT performances to detect Delta and Omicron variants were similar to the corresponding tests in a previous evaluation preceding the Delta/Omicron phase of the pandemic [11,12]. Three RDT (#11, #15 and #16) displayed impaired performance to detect antigen in Omicron specimen pools when compared with pre-Omicron/Delta pools of the previous evaluation (Figure 1) [11,12]. While this reduced performance of RDT #15 and #16 was observed for both Delta and Omicron specimen pools, the performance of RDT #11 to detect Delta was comparable to its performance with pre-Omicron/Delta variants. Moreover, three RDT (#1, #2, #5) showed impaired performance in the detection of Delta specimen pools, but not Omicron when compared with the pools in the previous evaluation (Figure 1). The remaining 14 RDT (#3, #4, #6–10, #12–14, #17–20) displayed either no difference or even enhanced performance to detect Delta and/or Omicron specimen pools when compared with the previous evaluation [11,12] (Figure 1). 

Cq: quantification cycle (here for E-gene assay); gc: genome copies; RDT: rapid antigen detection test; SARS-CoV-2: severe acute respiratory syndrome coronavirus 2.

RDT device and brand names not disclosed.

**Figure 2:**
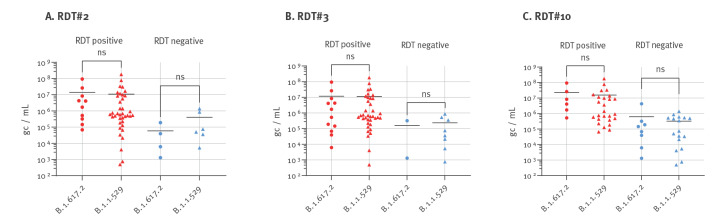
Comparison of rapid antigen detection tests of SARS-CoV-2 Delta and Omicron using non-pooled clinical specimens, Germany, January 2022

We also tested whether the use of pooled clinical specimens could have an effect on detection of the SARS-CoV-2 Delta and Omicron variant. Therefore, we randomly selected three RDT from the 20 tests included in this study and tested them with fresh, non-pooled clinical Delta (n = 14) and Omicron (n = 43) specimens ranging respectively from 1.29 × 10^6^ to 9.37 × 10^10^ and from 4.88 × 10^5^ to 1.81 × 10^11^ genome copies/mL. The average antigen detection at a given viral load using non-pooled samples was approximately equal for Delta and Omicron samples, while the spread was wider with Omicron than with Delta, potentially because more samples were used (Figure 2).

gc: genome copies. ns: not significant; RDT: rapid antigen detection test; SARS-CoV-2: severe acute respiratory syndrome coronavirus 2.

Fourteen Delta (B.1.617.2) and 43 Omicron (B.1.1.529) fresh, non-pooled clinical SARS-CoV-2 specimens were tested with three RDT selected from the list of 20 RDT evaluated in this study. Before testing, SARS-CoV-2 lineages were confirmed either by variant typing using commercial PCR or whole genome sequencing. y-axis: virus RNA genome copies/mL detected at the antigen-positive cut-off; x-axis: specimens of the different SARS-CoV-2 lineages; horizontal bars: means. Unpaired t-test was used for the statistical analysis.

## Discussion

While our data are in line with findings from several groups using clinical specimens or cell culture-derived virus [3-8], other groups have reported inferior detection of the Omicron variant by RDT compared with other variants [9,15]. Preliminary studies by the National Institutes of Health's RADx programme have suggested that RDT detect the Omicron variant in patient samples with reduced sensitivity [15]. However, when heat-inactivated patient samples were used, this did not result in reduced RDT sensitivity for Omicron [15]. Similarly, a study by Bekliz et al. initially reported that some RDT had significantly lower sensitivities for Omicron than for Delta, but the same authors later performed a retrospective study using clinical samples and found that all tested RDT showed comparable sensitivities for both variants [10,16]. Osterman et al. reported impaired detection by several RDT for Omicron in comparison with Delta when using respiratory swabs collected from different sampling sites stored at 2–8 °C for up to 1 week and frozen samples that were stored more than 7 days at −20 °C which were again thawed before antigen testing [9]. Based on our experience, antigen stability and RDT results are noticeably impacted by storage conditions (e.g. during transport) as well as additional freeze-thaw cycles. Specifically, we observed that the intensity of RDT test bands was reduced across our specimen panel upon additional freezing and longer storage periods (> 7 days) at 4 °C, consistent with a degradation of antigen as determined by quantitative antigen enzyme immunoassay (data not shown). Hence, controlled and uniform storage conditions for all samples used are critical for data interpretation and conclusions. Interestingly, Osterman et al. did not observe variant-dependent differences in RDT performance when cell culture-propagated isolates were used; for some RDT, they even saw better detection of Omicron, similar to our observations [9].

A decline in clinical performance of SARS-CoV-2 RDT could be caused by other factors than inherent properties of the currently predominant virus variants. In a recent pre-print, Meiners et al. argue that a reduction in clinical sensitivity of RDT observed from late 2020 to early 2022 among hospital employees in Berlin could instead be caused by the immunisation status of individuals [17]. By the end of the study, most individuals who presented for testing had either been repeatedly vaccinated or recovered from infection [17]. It is hypothesised that immunised individuals display faster immune reactions, resulting in an earlier onset of symptoms and therefore will present for testing earlier during the course of infection than naïve individuals [17]. At this stage, highly sensitive real-time PCR assays might identify infected individuals, while shedding of viral antigens might not yet be sufficient for the detection by an RDT [17,18].

According to a report by the European Centre for Disease Prevention and Control in January 2022 by the end of sampling for our study, BA.1 was the dominant Omicron sub-lineage in Germany (62.5%) [19]. At the same time, the BA.2 and BA.3 sub-lineages made up only 2.2% and 0.02%, respectively, of the uploaded Omicron sequences worldwide [20]. Hence, in this study we did not investigate the impact of more recently emerged Omicron sub-lineages on RDT performance. While new mutations present in Omicron are mostly located in the spike protein, only a few are located in the N protein, which is used by the majority of RDT (> 98%) as target antigen [21]. Comparing mutations across Omicron sub-lineages based on currently available GISAID sequence information, Omicron BA.1 and BA.2 share the same four mutations (P13L, DEL31/33, R203K and G204R) in the N protein, with the exception of S412R only present in BA.2 [22,23]. Omicron sub-lineages BA.3 and BA.5 share identical mutations in the N protein with BA.2, while BA.4 presents an additional mutation at P151S [22,23]. Further studies are needed to elucidate whether these additional mutations have an impact on RDT performance.

There are several limitations to this study. Comparing RDT detection performance for the Delta and Omicron variants using fresh, non-pooled clinical specimens confirmed that the average antigen detection at a given viral load was similar between Delta and Omicron also in non-pooled samples. However, there was an RDT-specific viral concentration range in which samples were detected as either positive or negative. This indicates that even with fresh, non-pooled specimens, such evaluations show a degree of variability close to the detection limit that subsequently could affect measured test sensitivities. One reason for this variability could be that viral RNA concentrations are not an ideal proxy to accurately determine viral antigen concentrations and that concentration ratios might differ between samples. Using pooled specimens can help to reduce such effects between individual samples as does replicate testing and increasing sample size. Furthermore, some of the RDT evaluated in this study displayed a faint test line over a broad range of virus concentrations, a phenomenon we also observed in previous evaluations using pre-Delta/Omicron specimen pools. This could be due to specific differences in RDT design or to the subjective grading system that relies on the interpretation of test line intensity by operators. Moreover, while there seems to be a correlation between test line intensity and antigen concentration, it might not be entirely linear and might differ between RDT. We observed occasional reappearance of test lines at lower concentrations in a few RDT, especially those with overall lower sensitivities and faint test lines. Moreover, we did not perform replicate testing of specimen pools in this study, which could further promote variabilities in test results. Ultimately, by using pooled clinical specimens, we deviated from the manufacturer’s instructions for RDT testing in regard to sampling. Despite this, however, using a standardised test panel consisting of pooled clinical samples allows for more uniform testing conditions that enable direct comparisons between RDT.

Overall, RDT sensitivities determined with pre-Delta/Omicron variants were widely confirmed in this small study. We cannot exclude that variations in detection performance observed for individual tests could potentially also be attributed to false positive/negative RDT results due to unspecific or failed antibody binding, lot-to-lot variations between studies, variations in test band intensity close to the detection limit upon repeated testing or differences in handling/interpretation by operators.

## Conclusion

Delta and Omicron specimen pools were detectable by RDT at a comparable level in this study. Furthermore, the performance of most tested RDT to detect Delta and Omicron was similar as for pre-Delta/Omicron specimen pools. Using an exemplary selection of RDT covering a range of sensitivities, our findings suggest that currently available RDT are sufficient to detect SARS-CoV-2 Delta and Omicron variants.
